# The relationship between the accuracy of curling athletes’ duration judgment and delivery performance

**DOI:** 10.7717/peerj.13541

**Published:** 2022-06-14

**Authors:** Minjia Song, Qiwei Zhao, Chunhua Du, Chenglin Zhou, Ruitao Li

**Affiliations:** 1 Graduate School, Harbin Sport University, Harbin, Heilongjiang, China; 2 School of Psychology, Shanghai University of Sport, Shanghai, China

**Keywords:** Duration judgment, Neural efficiency, Sports experience, Delivery performance

## Abstract

**Objective:**

Time perception is a critical point for curling athletes to have in order to successfully complete interactions between themselves and their environment. Exploring the relationship between the accuracy of duration judgment and curling athletes’ performance is helpful to reveal the influencing factors on their performance and to provide a reference for the training of athletes’ delivery performance.

**Methods:**

Thirty curling athletes and 30 non-athletes were recruited as participants. Using 3D modeling technology, curling videos of different situations were presented to the participants as stimulus information, and the participants were required to complete the duration judgment task. The neural activation of the participants during the entire process of duration judgment was recorded using electroencephalogram (EEG) equipment. The performance of the 30 curlers participating in the experiment was measured. Variance analyses were conducted on the collected behavioral and EEG data, and correlation and regression analyseswere conducted between behavioral data and delivery performance.

**Results:**

The accuracy of the distance judgment of curlers was higher than that of non-curlers (*P* < 0.05). In the stimulus video presentation stage, the power in the alpha band of curlers was higher than that of non-athletes (*P* < 0.05). In the task decision stage, the power in the alpha band of curlers was higher than that of non-athletes (*P* < 0.05), and the power in the theta band was higher than that of non-athletes (*P* < 0.05). There was a correlation between the accuracy of the curlers’ perception of specific situational time intervals and the accuracy of delivery (*P* < 0.05). Regression analysis results were y = 3.422 + 1.415x.

**Conclusion:**

The accuracy of curling athletes’ duration judgment is high in a specific situation. There is a correlation between the accuracy of duration judgment and delivery performance in a specific situation: the higher the accuracy of specific duration perception, the higher the performance accuracy of delivery. The cognitive strategies adopted by curlers differ from those adopted by non-athletes in the completion of duration judgment. Specifically, in a specific situation, fewer attention resources are utilized in the stimulus presentation and decision-making stages, while more memory resources are utilized in the decision-making stage to ensure higher accuracy of interval judgment. This study provides a new idea for exploring the causes of curling athletes’ excellent technical performance and provides a reference for future curling research on competition training practice. Given the limitations of mobile EEG devices in this study, future studies can measure neural activity during actual delivery preparation and execution in an environment of high ecological validity to obtain more direct evidence.

## Introduction

Many studies have shown that high-level athletes are not only elite in the field but also perform better than non-athletes in laboratory cognitive tests, which also reflects the importance of cognitive skills in competitive sports (Abernethy & Russell, 1987; Hua et al., 2011). In the process of motor learning and control, the movement must be generated based on perception (Zhang, 2003). Accurate action not only reflects the biomechanical characteristics of body posture and movement output but also the cognitive processing of information input (Feng, 2020). For example, relevant studies on tennis and badminton have found that athletes’ performance is closely related to general cognitive power such as cognitive control, working memory, and selective attention (Chen, 2017). In curling, accurate delivery is key to victory. Delivery refers to the whole process of a curler (*e.g.*, pushing, gliding, releasing the stone, etc.). Athletes need to control the movement speed of the curling stone so it stops in the ideal position according to the expected route, which is collectively called the “performance of throwing”. Excellent performance is inseparable from accurate time perception.

Ice reading is the basis of the delivery, which refers to the process of understanding and mastering the ice surface (*e.g.*, its smoothness). From the perspective of cognitive psychology, we can understand ice reading as the duration of curling motion. Therefore, it is of certain significance to explore the relationship between the delivery performance and the perception of duration.

The perception of duration is a type of time perception, including the estimation of time and the prediction and preparation of event occurrence time (Thones & Stocker, 2019). Duration refers to estimating the duration of an event’s existence (Peng, 2012). Studies have shown that athletes in different sports have different characteristics of duration. For example, the accuracy of duration estimation of divers was higher when facing the stimulus of diving action than novice (Liang, Zhang & Zhang, 2018). The duration replication of high-level pole vaulters is more accurate and more stable (Chen, Pizzolato & Cesari, 2014).

In the organization and execution of the whole delivery movement, curling athletes need to constantly perceive and evaluate their curling movement and judge whether they can reach the target position according to the established route and speed to adjust the movement. Therefore, there may be a close relationship between the accuracy of curling athletes’ duration judgment and their performance.

Athletes’ cognitive skills are improved in the long-term training process, especially when the test content is related to the sport-specific situation. This cognitive advantage is more obvious, and sports performance can be expected to a certain extent (Chun-Hao, David & Shih-Chun, 2019). When athletes completed a prediction task in different situations, the video prediction response was slower, and the accuracy was higher than that of picture prediction. The possible mechanism is that the cortical activation duration was longer during cognitive processing, and the psychological resources were less in the early stage and more in the late stage (Zhao & Guan, 2014). In curling, the marking line may be an important situational factor affecting athletes’ judgment of duration. The mark line of the field is important for reminding athletes, as the hog line of the delivery area is important for reminding athletes when to release, and the house is important for reminding them of the strength and direction of sweeping. Thus, it can be speculated that when the available reference information is matched with one’s own experience, athletes can efficiently complete the processing and extraction of basic information and call psychological resources into higher-level cognitive processing to complete accurate judgment. Some studies have also found that athletes’ cognitive advantages exist only in professional-related cognitive tests (Mori, Ohtani & Imanaka, 2002). Therefore, the relationship between curling athletes’ duration judgment performance and delivery performance and its cognitive mechanism are more likely to be reflected in special situations.

Athletes’ cognitive advantage is not only reflected in their behavioral performance but also in their specific neural activities (Wei, 2019). According to the neural efficiency hypothesis, long-term systematic sports training causes the structural and functional plasticity changes of athletes’ brains, leading to the improvement of neural efficiency (Dennis et al., 2013). The application of an electroencephalogram (EEG) provides an effective way to explore the neural efficiency characteristics of athletes. For example, compared to inexperienced novices, the alpha power of professional athletes is lower in the preparation and execution of golf putting, reflecting the efficiency of attentional resource invocation (Urgen et al., 2013). The alpha spectral (10–13 Hz) power in the left temporal lobe of shooters is higher than that in the right, which is related to the reduction in attentional resource consumption, reflecting the automatic execution of shooting actions when high-level athletes suppress visual attention (Baumeister et al., 2008). The cognitive superiority of duration judging allows curlers to automatically complete the task with fewer attentional resources. Therefore, curlers may outperform novices while having a higher alpha power, reflecting the lower neural recruitment, which aligns with the neural efficiency hypothesis. Another common electrophysiology indictor to estimate the athlete cognitive advantages is theta band power, which is often considered to be related to the encoding and recall of episodic memory as well as cognitive conflict and workload (Kahana et al., 1999; Loze, Collins & Holmes, 2001; Lega, Jacobs & Kahana, 2012). Curlers may present a greater theta power than novices when judging durations, which reflects the increased demand of specific cognitive functions such as episodic memory recall and cognitive control.

In conclusion, to explore the relationship between curling athletes’ duration judgment accuracy and their delivery performance, this study adopted the expert—novice paradigm and EEG data to explore the behavioral characteristics of curling athletes’ duration judgment accuracy and cerebral cortex activation characteristics by setting the duration judgment task of situational factors. This study makes the following assumptions: (1) curling athletes’ duration judgment has cognitive advantages compared to non-athletes and is subject to specific situational adjustment, (2) the accuracy of curling athletes’ duration judgments is related to their delivery performance, and (3) curlers show neural efficiency in duration judgment.

## Materials & Methods

### Characteristics of curling athletes’ duration judgment accuracy

#### Participants

G*Power 3.1 was used to calculate the required number of participants. With the power of 0.8, the required number of participants was 26 in each group. We recruited 30 professional curlers as participants (17 men and 13 women; mean age = 21 ± 5.33 years; mean height was 173.63 ± 6.39 cm). These professional curlers have at least 3 years of professional curling experience. In most cases, the curlers train six hours a day, five days a week. Of these 6 h, there are 2 h of ice training and 2 h of land simulation. In addition, 30 college students were recruited as non-athletes (16 men and 14 women; mean age = 20.63 ± 1.22 years; average height = 71.37 ± 7.20 cm). This group did not have any special sports experience or sports habits. All participants were right-handed, had normal or corrected-to-normal vision, did not consume alcohol, were in good physical condition, and had no known mental disorders.

#### Experiment design

The duration judgment experiment adopted an experimental design of two participants (sports experience: curlers, non-athletes) × two participants (application situation: general situation, special situation), and the independent variables were sports experience and application situation. The dependent variables include the absolute error value (AEV), that is, the absolute value of the judgment error value where judgment error value = judgment value − accurate value. Theta band power and alpha band power. This experiment was approved by the Ethics and Ethics Committee of Harbin Sport University (No. 2022001).

#### Stimulus material

In the experiment, 4D-CINEMA R 20.0 (Maxon Computer, Friedrichsdorf, Germany) was used to produce a 3D virtual curling video as stimulus material. The video resolution was 1920 ×1080 pixels, and the frame rate was 30 frames/SEC. The height of the virtual camera was 170 cm and it was located 9 m in front of the hog line in the competition area. The focal length was 50 mm and the initial angle of view was 40.36°, which simulated the horizontal vision of the human eye. Under general conditions, the picture shows a curling, red line, and green line. Under special conditions, the screen presents curling, hog lines, and houses. To avoid the repeated use of the same experimental material, which would lead to the learning effect of the participants, we adjusted the movement track and curling speed. From appearance to the disappearance, the curling stone moved in a straight line at an initial speed of 14.25°s. Curling had 10 different starting points. The video was processed at 0.6, 0.7, and 0.95 times speed, and the video length was 2866 ± 400 ms (Table 1).

**Table 1:** Stimulus material details.

Application situation	Video content	Speed of movement	Total quantity
general situation	White ice, a curling, red line, and green line	the curling stone moved in a straight line at an initial speed of 14.25°s.	60
special situation	Standard curling court, a curling, hog lines, and houses		

**Figure 1:**
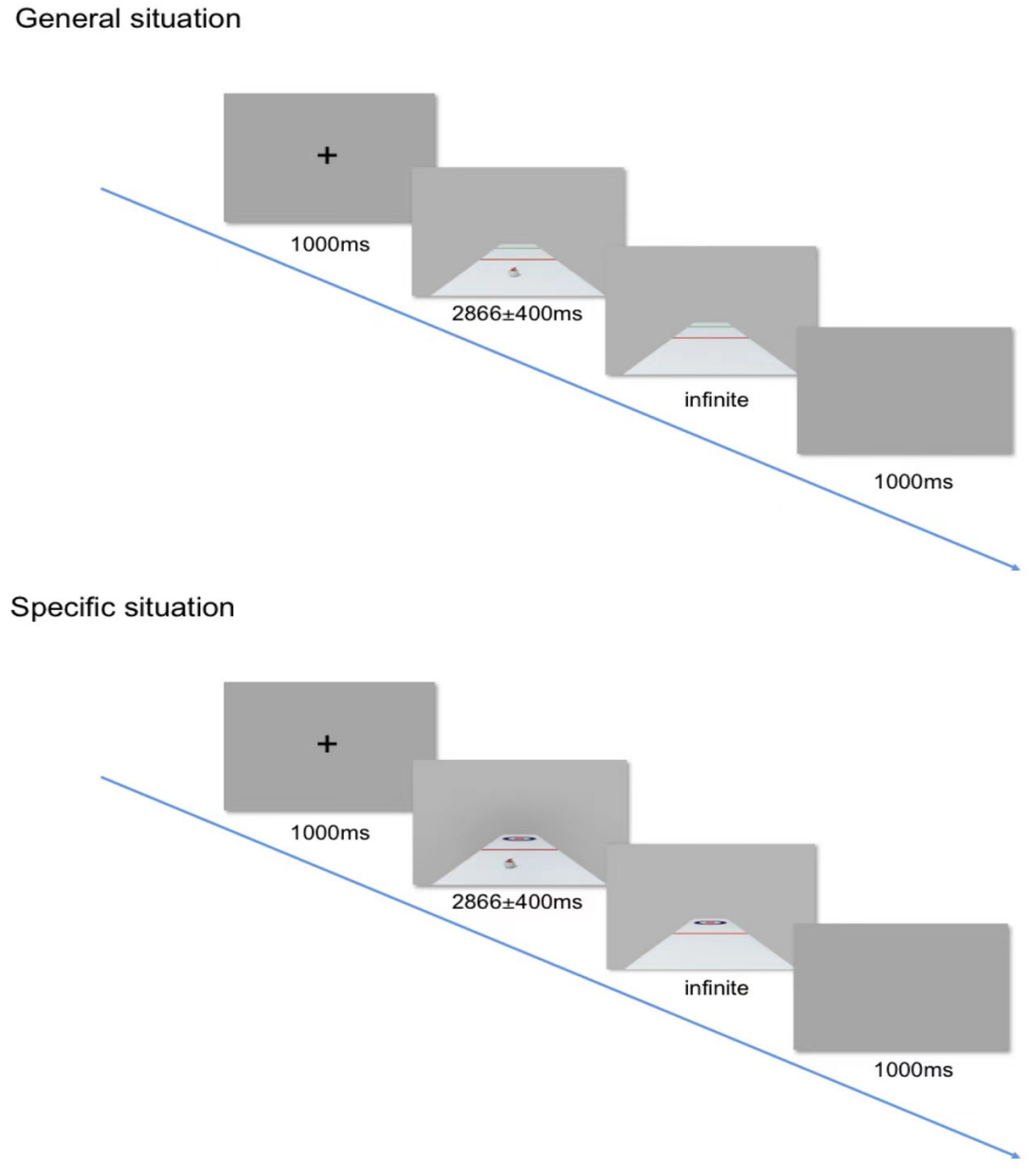
The experimental program.

After entering the laboratory, each participant first filled out an informed consent form and a participant information collection form. Before the experiment began, the experimenter explained the experimental content and task and practiced the experiment. After the participants mastered the experimental tasks, the practice trial ended and the formal experiment began. Combined with curling practice, a duration judgment task was designed. The task required the participants to watch a curling video. The curling stone in the video moved to the red line with a certain movement pattern, entered the covered area, and disappeared. Curling continues on the same schedule as before and disappears. The participants were asked to judge the time when the front end of the curling stone touched the T line or the green line and responded to the button. All participants completed all tests under general conditions and all tests under special conditions (Fig. 1).

#### Experimental apparatus

In this experiment, Quick-20, a 23-channel EEG recording device produced by the CGX Company in the United States was used to collect EEG data. Sensors are in an international standard 10–20 layout with a total of 23 electrode positions (20 channels + reference wires + 2 ground electrodes). The common reference point was obtained using an ear clip electrode at a sampling rate of 1,000 Hz. Two desktop computers, a Pentium IV CPU, Windows 10 operating system. One was used to present stimuli and record behavioral data, and the other was linked to the electrical brain equipment and recorded electrical brain data. The display was a 22-inch (1024 ×768 resolution, refresh rate of 100 Hz) Lenovo LCD. The presentation of experimental stimuli and the recording of behavioral data were completed using E-Prime 3.0.

#### Data acquisition and processing

Behavioral data were collected using E-Prime 3.0. The extreme values were removed. Two invalid data points were removed from the two groups (*N* = 28). SPSS 18.0 was used to conduct a two (sports experience: curler, non-athlete) ×2 (application situation: specific situation, general situation) repeated-measures analyses of variance (ANOVA) for behavioral data, and the analysis index was AEV. The smaller the AEV value, the more accurate the speed perception.

EEG data was acquired by CGX Acquisition 6.4, and after data inspection and extreme value removal, two invalid data were removed from the two groups respectively, at which *N* = 28. The Brain Production Analyzer 2.1 software was used for preprocessing. The pretreatment steps mainly include removing useless channels and reducing the sampling rate, filtering (50 Hz, high pass 0.1, low pass 60 Hz, slope 24 Bb/OCT), and the method of Gratton and Coles was used to remove the electro ophthalmology. Segment: start the video 1,000 ms, segment the video 1,000 ms ms after the end, and average the EEGs of this period. Finally, the signals under the two-time windows were processed by FFT. The analysis area was the prefrontal area: Cz and Fz; occipital lobe: O1 and O2. The selected components were the alpha band (8–15 Hz) and theta band (4–8 Hz). For the power of the two-time windows of the above two frequency bands, an ANOVA of repeated measures of two (sports experience: curler, non-athlete) ×2 (application situation: general situation, specific situation) was conducted. The analysis index was the power of each frequency band.

### Delivery performance measurement

#### Participants

The delivery performance of 30 curlers in the duration judgment task was measured.

#### Measurement scheme

To measure the performance of curling athletes, we used the fixed-point method. According to curling competition needs, we chose the centerline and the house of the intersection of eight points as the shooting point. With each drop point as the center of the circle and the length of the diameter of the stone as the diameter, the resulting circle is denoted as C1. With each drop point as the center of the circle and the length of the diameters of the two stones as the diameter, the resulting circle is denoted as C2. Five circles are used for each spot. Each participant threw the pot five times with the goal of throwing it on time. Five points were scored if curling stopped in C1, and four points were scored if curling stopped in C2. The curling stops at C5 and scores one point. Not in range C1-C5, no score.

#### Data processing

First, discard invalid data. Because there were outliers in the data of the two athletes in the time-distance judgment task, the data of their delivery performance were not counted in the processing of their performance data. First, the average score of the delivery performance of the remaining 28 participants was calculated. The average value was used as the index to evaluate delivery accuracy. The higher the accuracy index, the higher the accuracy level. Then, the behavior data of 28 curlers in general and special situations in the time distance judgment task were correlated with the mean value of the delivery performance score. Then, the behavioral data of 28 curlers in general and special situations in the duration judgment task were analyzed with correlation and regression analyses with the mean value of the delivery performance score.

## Results

### Behavioral result of duration judgment

**Figure 2:**
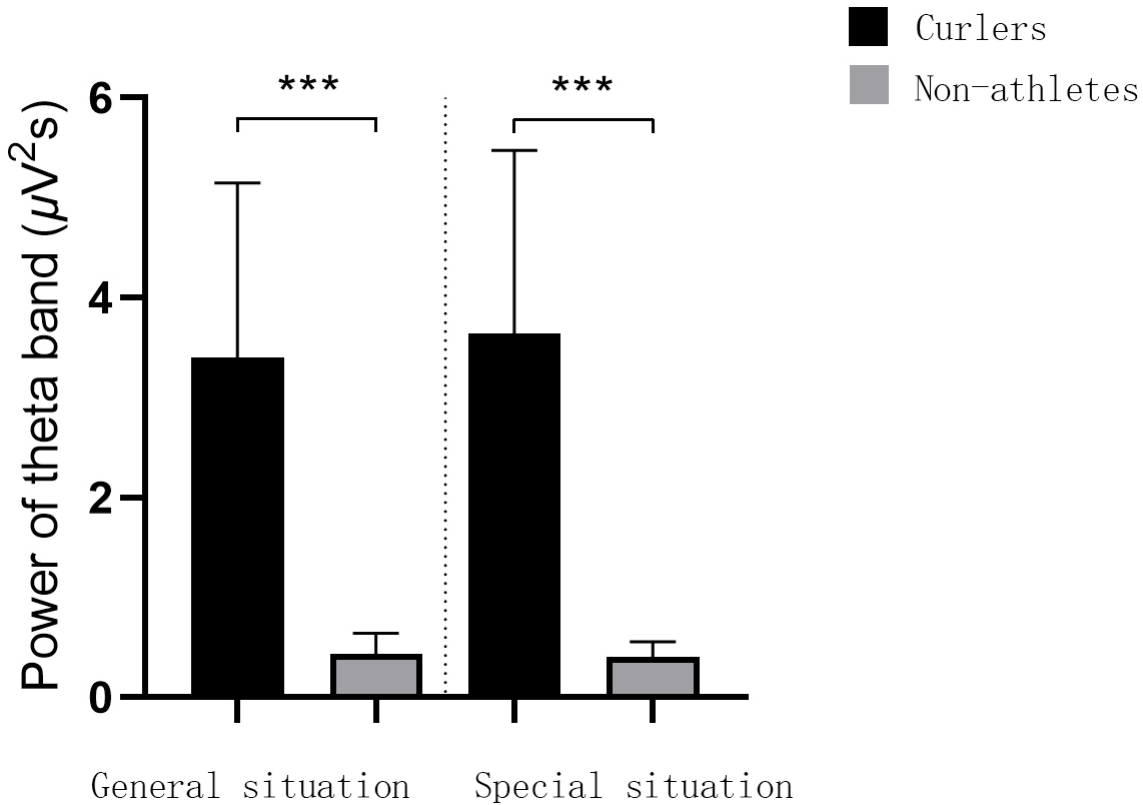
The behavioral result of duration judgment. Three asterisks (***) denote *p* < 0.001.

ANOVA results of repeated measures of two (sports experience: curler, non-athlete) ×2 (application situation: general situation, specific situation) of duration judgment behavioral data showed that the main effect of sport experience F(1,27) =24.29, *p* = 0.000, *η*^2^_*p*_ =0.47, application F(1,27) = 6.66, *p* = 0.016, *η*^2^_*p*_ = 0.20, the interaction between application situation and sports experience F(1,27) = 4.34, *p* = 0.047, *η*^2^_*p*_ = 0.14 and were all significant. The results of the simple effect analysis showed that the absolute error value of duration judgment between curlers and non-athletes was significant in general situations *p* = 0.03, 95% IC [36.03–559.81], and the absolute error value of curlers (1142.53 ± 43.89) was significantly smaller than that of non-athletes (1440.44 ± 124.91). In the special situation, the absolute error values of curling athletes and non-athletes were significant *p* = 0.000, 95% IC [405.55–1237.05], and the absolute error value of curlers (552.20 ± 38.86) was significantly smaller than that of non-athletes (1373.50 ± 203.48). There was a significant difference in the absolute error value of curling athletes’ duration judgment in general and special situations *p* = 0.000, 95% IC [450.43–730.22], and the absolute error value of curlers in general situations (1142.53 ± 43.89) was significantly greater than that in special situations (552.20 ± 38.86). The absolute error value of the duration judgment of non-athletes in general and special situations was different, but not significant *p* = 0.786, 95% IC [432.84–566.72]. The absolute error value of non-athletes in general situations (1440.44 ± 124.91) was greater than that in special situations (1373.50 ± 203.48) (Fig. 2).

The results show that curlers can distinguish between two different application situations well, and the accuracy of duration judgment in specific situations is higher than that in general situations. However, non-athletes cannot distinguish between the two different application situations.

### Cortical activation characteristics of duration judgment

**Figure 3:**
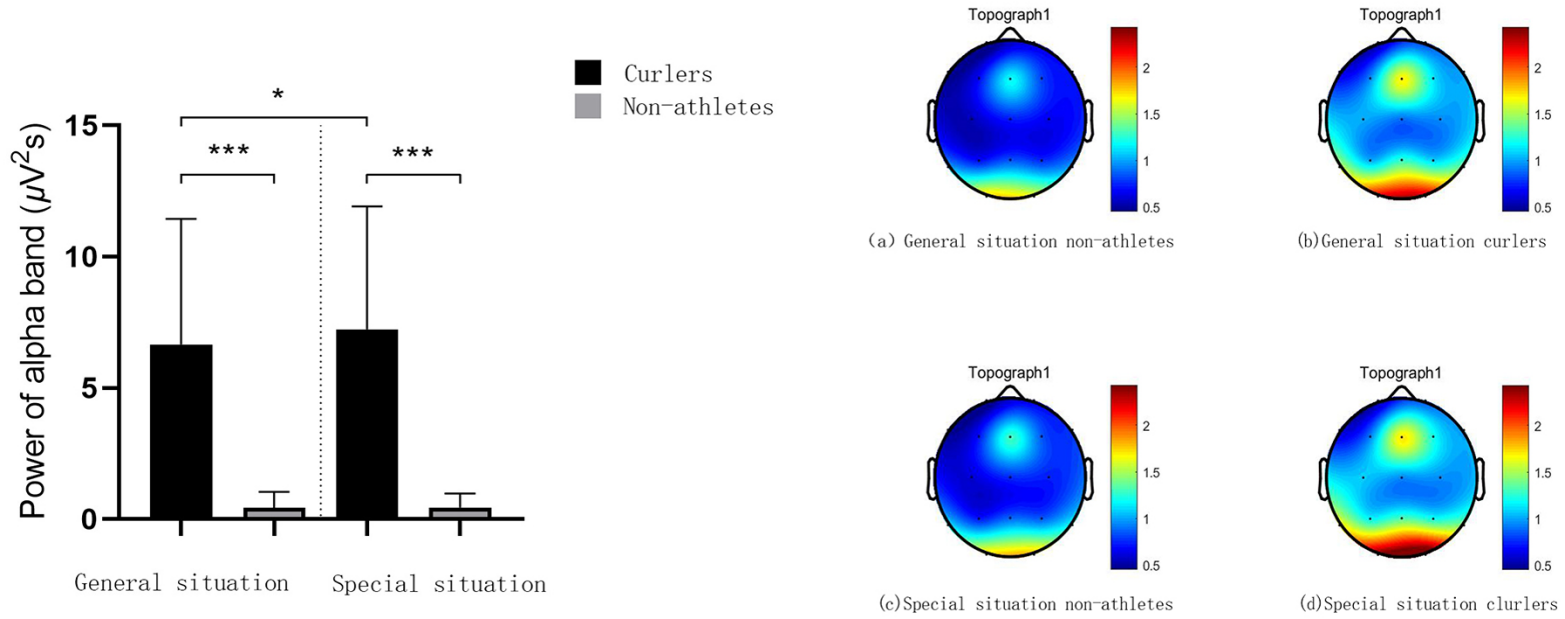
Alpha frequency power value and spectrum energy in T1 time window. The bar graph of alpha frequency power value in T1 time window is on the left side of the picture. The alpha spectrum energy diagram of T1 time window is on the right of the picture. An asterisk (*) denotes *p* < 0.05, three asterisks (***) denote *p* < 0.001.

The results of repeated measures ANOVA of duration judgment EEG data showed that the main effect of sport experience was significant in the T1 time window F(1,27) = 53.46, *p* = 0.000, *η*^2^_*p*_ = 0.66, the main effect of application situation was significant F(1,27) = 6.21, *p* = 0.019, *η*^2^_*p*_ = 0.19, and the interaction between application situation and sports experience was significant F(1,27) = 5.91, *p* = 0.022, *η*^2^_*p*_ = 0.18. The simple effect analysis results showed that, in general, the alpha band power of curlers and non-athletes was significantly different under the T1 time window *p* = 0.000, 95% IC [4.36–8.07], and the alpha band power of curlers(6.65 ± 0.91) was significantly greater than that of non-athletes (0.44 ± 0.12). In the special situation, the alpha band power generated by curlers and non-athletes in the T1 time window was significantly different *p* = 0.000, 95% IC [4.96–8.61], and the alpha band power generated by curlers (7.23 ± 0.89) was significantly greater than that generated by non-athletes (0.44 ± 0.10). For curlers, under the T1 time window, there is a significant difference in the alpha band power generated under the general situation and the special situation *p* = 0.019, 95% IC [0.10–1.06], and the alpha band power generated under the general situation (6.65 ± 0.91) is significantly smaller than that under the special situation (7.23 ± 0.89). For non-athletes, in the T1 time window, there was no significant difference in the alpha band power generated under general situational stimuli and specific situational stimuli *p* = 0.904, 95% IC [0.06–0.07], and the alpha band power generated under general situational stimuli (0.44 ± 0.12) was smaller than that under specific situational stimuli (0.44 ± 0.10). In addition, repeated measure ANOVA results showed that there was no significant difference in the theta band power under the T1 time window (Fig. 3).

**Figure 4:**
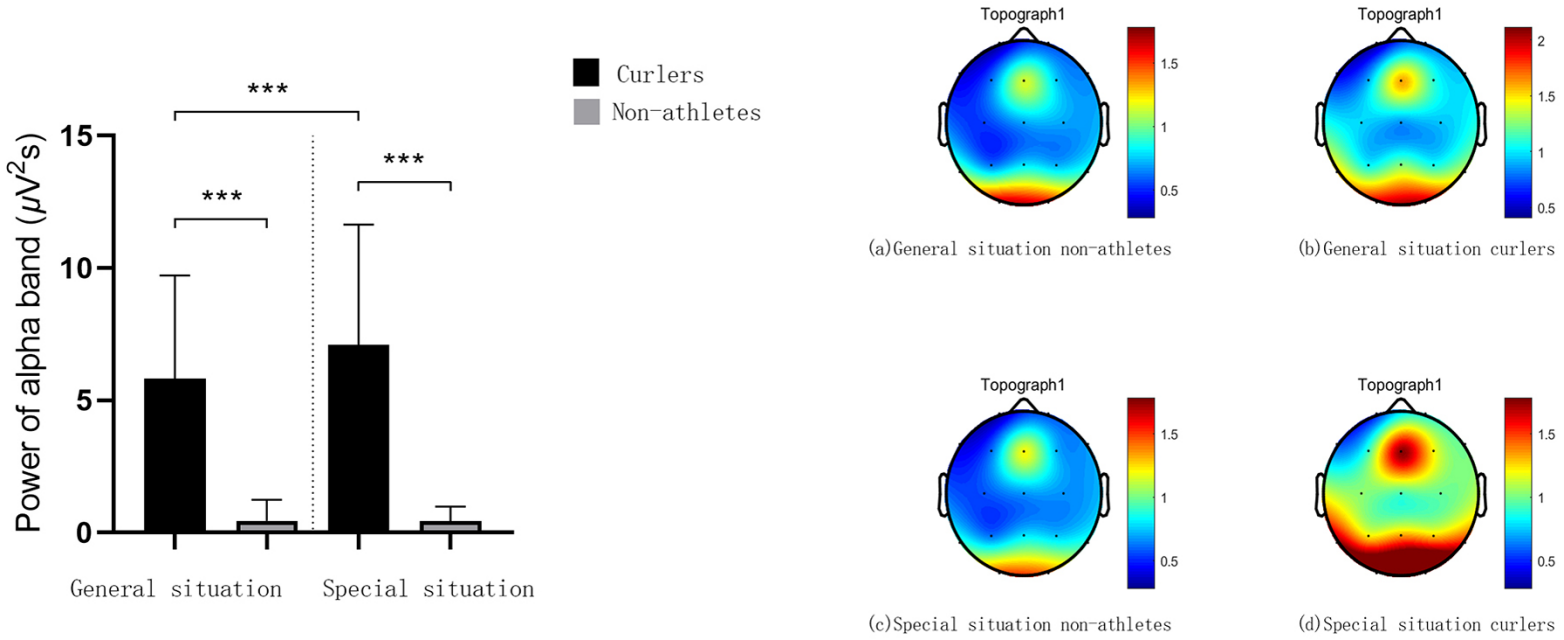
Alpha frequency power value and spectrum energy in T2 time window. The bar graph of alpha frequency power value in T2 time window is on the left side of the picture. The alpha spectrum energy diagram of T2 time window is on the right of the picture. Three asterisks (***) denote *p* < 0.001.

Repeated measure variance analysis of duration judgment EEG data showed that, under the T2 time window, the statistical results of alpha frequency power showed that the main effect of sport experience was significant F(1,27) = 57.11, *p* = 0.000, *η*^2^_*p*_ = 0.68, the main effect of application situation was significant F(1,27) = 24.50, *p* = 0.000, *η*^2^_*p*_ = 0.48, and the interaction between application situation and sports experience was significant F(1,27) = 22.94, *p* = 0.000, *η*^2^_*p*_ = 0.46. The results of the simple effect analysis showed that, in general, the alpha band power generated by curlers and non-athletes was significantly different in the duration judgment stage *p* = 0.000, 95% IC [3.84–6.90], and the alpha band power generated by curlers (5.82 ± 0.74) was significantly greater than that of non-athletes (0.45 ± 0.15). In a special situation, the alpha band power generated by curlers and nonathletes is significantly different in the duration judgment stage *p* = 0.000, 95% IC [4.89–8.45], and the alpha band power generated by curlers (7.10 ± 0.86) is significantly greater than that generated by nonathletes (0.43 ± 0.10). There is a significant difference in the alpha band power generated by curling athletes in general situations and in special situations when judging the duration *p* = 0.000, 95% IC [0.76–1.80]. The alpha band power generated in the general situation (5.82 ± 0.74) was significantly smaller than that generated in the special situation (7.10 ± 0.86). There was no significant difference in the alpha frequency band power generated by non-athletes in general and special situations *p* = 0.777, 95% IC [0.11–0.15]. The alpha frequency band power generated in the general situation (0.45 ± 0.15) was greater than that generated in the special situation (0.43 ± 0.10) (Fig. 4).

**Figure 5:**
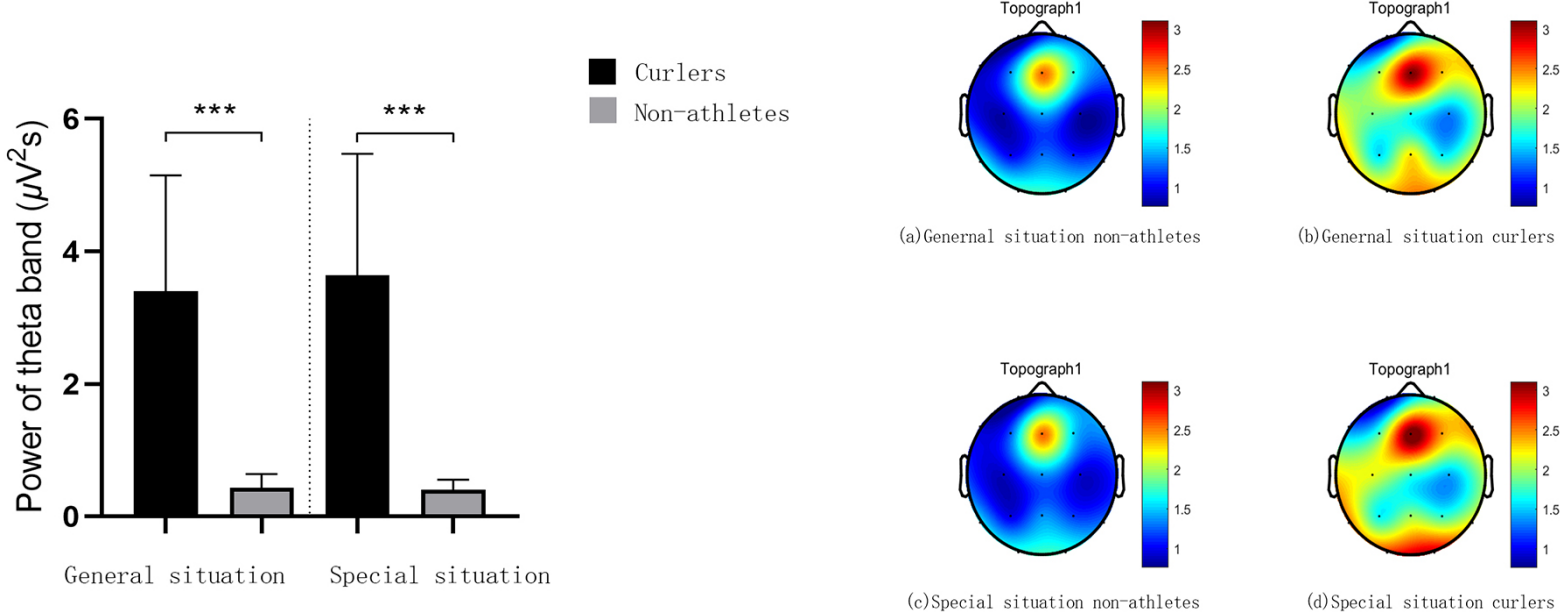
Theta frequency power value and spectrum energy in T2 time window. The bar graph of theta frequency power value in T2 time window is on the left side of the picture. The theta spectrum energy diagram of T2 time window is on the right of the picture. Three asterisks (***) denote *p* < 0.001.

The results of repeated measures variance analysis of duration judgment EEG data showed that under the T2 time window, the statistical results of theta band power showed that the main effect of sport experience was significant F(1,27) = 89.64, *p* = 0.000, *η*^2^_*p*_ = 0.77, and the application situation had a significant interaction with sports experience F(1,27) = 4.97, *p* = 0.034, *η*^2^_*p*_ = 0.16. The simple effect analysis results showed that, in general, the theta band power generated by curlers and non-athletes was significantly different in the T2 time window *p* = 0.000, 95% IC [2.30–3.63], and the theta band power generated by curlers (3.40 ± 0.33) was significantly greater than that generated by non-athletes (0.43 ± 0.04). In this special situation, the theta band power generated by curlers and non-athletes is significantly different in the T2 time window *p* = 0.000, 95% IC [2.54–3.94], and the theta band power generated by curlers (3.64 ± 0.35) is significantly greater than that generated by non-athletes (0.40 ± 0.03). For curlers, in the T2 time window, the difference between the theta band power generated in the general and specific situations was significant *p* = 0.078, 95% IC [0.03–0.50], and the theta band power generated in the general situation (3.40 ± 0.33) was smaller than that in the specific situation (3.64 ± 0.35). For non-athletes, in the T2 time window, the difference between the theta band power generated in the general and specific situations was significant *p* = 0.072, 95% IC [0.00–0.07], and the theta band power generated in the general situation (0.43 ± 0.04) was greater than that in the specific situation (0.40 ± 0.03) (Fig. 5).

The results showed that under the T1 time window, the alpha band power of curlers in the special situation was significantly higher than that in the general situation, and the alpha band power of curlers was significantly higher than that of non-athletes. However, there was no difference in the alpha band power of non-athletes in the different situations. Under the T2 time window, the theta band power of athletes was significantly higher than that of nonathletes in a specific situation. In addition, the alpha band power of athletes in the special situation was significantly higher than that of athletes in the general situation.

### Correlation analysis between delivery performance and duration judgment accuracy

**Figure 6:**
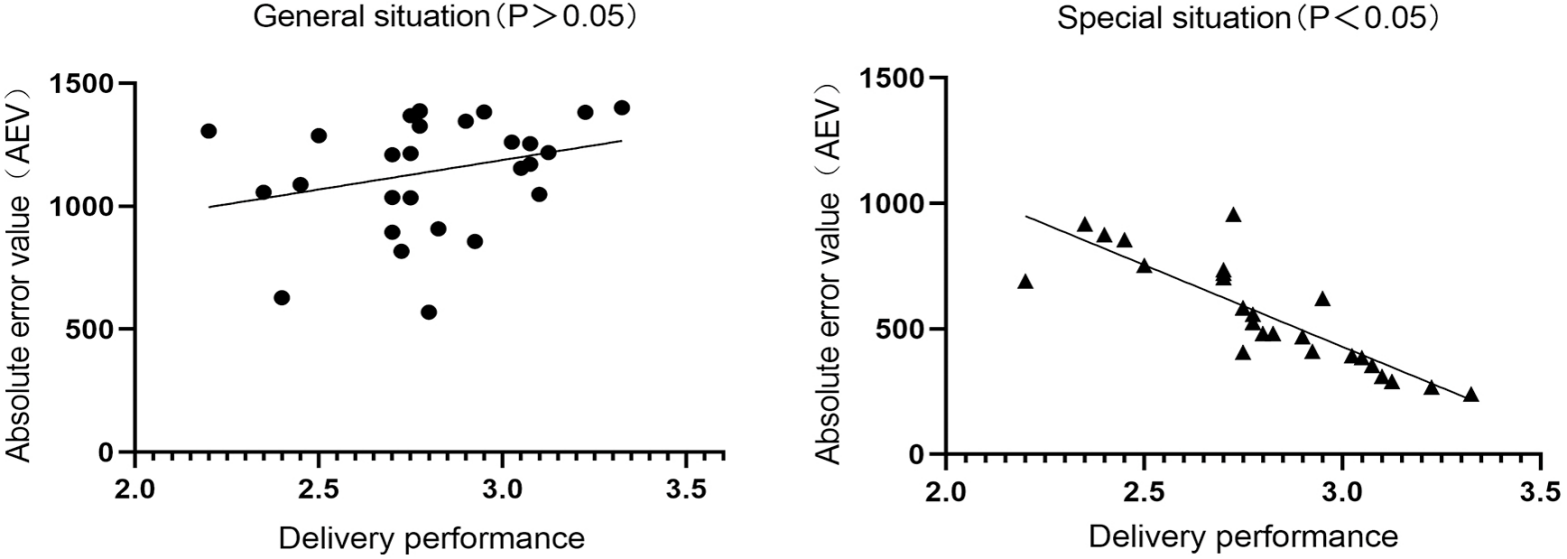
The relationship between accuracy of duration judgment and delivering performance.

The results of the correlation analysis show that the AEV of duration judgment was not correlated with the average value of delivery performance in general situations (*r* = 0.277, *P* = 0.154), while the AEV of duration judgment is highly negatively correlated with the average value of delivery performance in special situations (r = −0.849, *P* < 0.000). A regression analysis was conducted on the absolute error of duration judgment and the average value of delivery performance under special situation. When the absolute error of duration judgment was taken as the independent variable and the average value of delivery performance was taken as the dependent variable, there was a correlation between the two. The regression constant was 3.422, the regression coefficient was −0.001, and the regression equation was *y* = 3.422 + 1.415*x* (Table 2 and Fig. 6).

## Discussion

It can be seen from the results of the duration judgment task that compared to non-athletes, curlers have higher accuracy in duration judgment, but this expert cognitive advantage is more obvious in special situations. This finding is consistent with the empirical hypothesis of experts’ cognitive advantage. Long-term competitive training improves athletes’ cognitive skills (Raghavachari, 2006). Many studies believe that elite athletes in the field of sports perform better than non-athletes in perception, expectation, and decision-making, and their cognitive advantages are more prominent in tasks related to their sports expertise, which are reflected in attention, memory, decision-making, and other aspects (Chen & Cesari, 2015). It is worth noting that the regulation effect of specific situations on the accuracy of duration judgment only exists in curlers. This finding is consistent with the results of previous studies. When the exercise expert participants were asked to complete the duration replication task under different stimuli, the expert participants performed better under the professional field-related stimuli than the general stimuli. It was believed that professional experience improved the accuracy of action expectation (He et al., 2018), which further illustrates the importance of specific situational information in athletes’ duration judgment, and the situational information in tasks affects how individuals extract information to guide their behaviors (Chun-Hao, David & Shih-Chun, 2019). When special information such as a house or hog line is presented, curlers can make use of it more effectively and further control their behavior. Therefore, the judgment accuracy of the curling duration was higher than that in the general situation without information. For non-athletes, due to a lack of curling-related sports experience, they cannot extract effective reference information in specific situations.

**Table 2:** A descriptive statistical table of delivering performance.

Samples	The average delivering time	Total delivering time	Total points	The average points (M ± SD)
28	40	1120	3148	2.811 ± 1.068

Combined with the actual field performance test, the correlation analysis revealed a relationship between curling athletes’ duration perception and their performance. The more accurate the curling athletes’ judgment of duration, the better their performance. The relationship between athletes’ duration judgment and delivery performance can be described by linearly. As mentioned above, the movement must be generated based on perception. The execution of delivery is based on the result of duration perception, and high-precision duration perception is the foundation of high delivery performance. According to the theory of motor learning and control, action generation can be regarded as an information processing process, which can be divided into three stages: stimulus information input, information processing, and action information output (Zhang, 2003). In the whole organizational process of the delivery, the curlers have experienced three orderly links: the collection of information on curling moving duration, the transformation and processing of action information, and the output of the delivering action. In this process, the cognitive advantage of curling athletes’ information processing is reflected in the judgment of duration, which affects the final motion output of the delivery movement. This is consistent with previous studies on the cognitive characteristics of athletes, such as tennis players’ speed perception, which is correlated with the accuracy of landing point of forehand and backhand strokes, and depth perception, which is correlated with the accuracy of landing point of overhand serves (Chen, Pizzolato & Cesari, 2013a; Chen, Pizzolato & Cesari, 2013b).

Condition evaluation and diagnosis are necessary links throughout the whole training process of athletes. It is a common operation in sports selection and training practice to predict or evaluate athletes’ performance by a certain physiological or psychological index (Thomas, Pellegrini & Holmberg, 2018). The results of evaluation and diagnosis are important basis for making and adjusting training programs. It can be inferred from the results of the regression analysis that when the accuracy of the athlete’s time perception reaches a certain value, their performance can be predicted to a certain extent. This model can provide some reference for the condition diagnosis of curlers. In addition, the correlation between duration judgment and delivery performance does not exist in general situations, which further illustrates the importance of situational reference information in the interaction between perception and action (Mori, Ohtani & Imanaka, 2002). Curlers’ sports experience can be used to a great extent in special situations, and their special cognitive strategies allocate cognitive resources reasonably.

In addition, EEG spectrum power analysis further revealed the cognitive advantages and neural efficiency of curling athletes’ duration judgments. In the stimulus presentation stage, the alpha power of curlers was significantly higher than that of non-athletes in both general and specific situations; however, there was no difference in the theta band. Previous studies on the neural efficiency hypothesis have found that higher alpha power reflects relatively lower consumption of neural resources, which is usually a manifestation of efficient spatial attention processing (Lu, 2020). Consistent with previous expert-novice comparisons in events such as putting, shooting, archery, and darts, expert athletes showed decreased cortical activity during task completion (Wang, 2020; Cheng et al., 2015; Cooke, 2013; Hatfifield, 2018). This indicates that curlers have neural efficiency in the cognitive processing of curling stimulus information and can encode and process the given stimulus more efficiently than non-athletes. Moreover, the regulation effect of specific situations on alpha power exists only in curlers, and the alpha power is higher in specific situations. Combined with the above discussion on the influence of special situation information on behavioral performance, hog lines, houses, and other information can be used as important references related to special experiences, which can call fewer attention resources during the processing of duration-related information, reflecting the characteristics of neural efficiency (Hatfifield et al., 2004).

However, in the task decision stage, the power characteristics of the curlers in the alpha band were similar to those in the stimulus presentation stage. The theta band showed expert cognitive characteristics at this stage. Regardless of the general or special situation, the theta power of curlers is significantly higher than that of non-athletes. Previous studies have shown that theta band power is an intuitive indicator of cognitive load and is closely related to cognitive control, memory encoding, recall, and other advanced cognitive functions (Klimersch, 1999; Jensen & Tesche, 2002; Babiloni et al., 2009; Sauseng et al., 2010). Consistent with previous studies, table tennis players and non-athletes had different powers in the theta band when they completed professional motion-related cognitive tasks, reflecting cognitive advantages related to conflict control and memory processes in uncertain situations (Gevins et al., 1997).

Some researchers believe that high-level athletes’ more accurate duration estimation and judgment ability is the result of long-term sports training, benefiting from plasticity changes in the sensory-motor system (Dayan & Cohen, 2011; Lu, 2017). Some researchers believe that their cognitive advantages come from the improvement of working memory, attention, and other advanced cognitive functions involved in duration judgment (Chen, Pizzolato & Cesari, 2014; Huang et al., 2018). The findings of this study support a balance between the two. Compared to non-athletes, curlers have more accurate duration judgment and higher alpha and theta powers, reflecting the difference in cognitive source allocation caused by sports experience (Chen, Pizzolato & Cesari, 2013a; Chen, Pizzolato & Cesari, 2013b). On the one hand, higher alpha power reflects curlers’ lower-level cognitive processes, such as visual-spatial attention and neural resource-saving in motor information processing, and is more obvious under specific situational information reference. On the other hand, higher theta power reflects the increased cognitive input of curlers in task decision-making and may involve the recall of higher cognitive functions such as episodic memory (Guy et al., 2016). Because of the flexible allocation of neural resources, curlers use fewer cognitive resources to complete the underlying cognitive processing and devote more cognitive resources to higher-level cognitive processing and decision-making, thus helping them to make more accurate duration judgments. In summary, it can be inferred that curling athletes show neural efficiency in duration judgment.

As a complex dynamic system, the structure and function of the brain can be repaired and reorganized dynamically under the influence of learning, training, experience, and other factors (Gao, Wang & Zhang, 2016). In recent years, from several functional imaging and EEG studies, we not only have an in-depth understanding of the neuronal mechanisms that change during motor skill acquisition, but also have a better understanding of the plasticity changes occurring in these nervous systems with the improvement of motor task practice (Ulrike & Regine, 2006; Howard & Lieberman, 2014; Wong, Chan & Mak, 2014). Based on the verification of the relationship between curling performance and duration, this study also expounds the cognitive advantage behind curling athletes’ high time judgment performance from the perspective of neural efficiency. This study provides a research basis for further exploring the relationship between sports training and brain plasticity, opens up ideas for training methods to improve athletes’ competitive performance, and provides references for sports selection and athlete diagnosis.

This study had some limitations. Although this study revealed the relationship between duration judgment and delivery performance, its neural activity characteristics were explored only to a certain extent. Another important limitation is the negative effects of motion artifacts on EEG measurements. Improving the performance of mobile EEG devices (*e.g.*, reducing artifacts and improving cold resistance) is crucial for relevant research. In the future, we can combine biomechanical research methods and means to further explore the neural mechanism and mechanical performance in the actual curling process, so as to provide more reference for curling competition training.

## Conclusions

The accuracy of curling athletes’ duration judgment is adjusted by special situations, which shows that the accuracy of curling athletes’ duration judgment is high in certain situations. There is a correlation between the accuracy of a specific duration and the delivery performance. The higher the perception accuracy of specific duration, the higher the performance accuracy of delivery. The cognitive strategies adopted by curlers differ from those adopted by non-athletes in the completion of duration judgment. In the specific situation, fewer attention resources are utilized in the stimulus presentation stage and decision-making stage, while more memory resources are utilized in the decision-making stage to ensure higher accuracy of duration judgment. This study reveals the potential relationship between pot-throwing performance and time distance perception, and provides a new method for improving pot-throwing performance. In this study, EEG data were recorded only in the duration judgment task (not in the process of delivery); thus, the neural activity in the actual delivery process could not be further explored. With the advancement of portable EEG or near-infrared technology, future studies can measure neural activity during the preparation and execution of actual delivery in a context of high ecological validity to obtain more direct evidence. Future studies should explore the specific impact of duration perception training n the improvement of delivery performance.

##  Supplemental Information

10.7717/peerj.13541/supp-1 Supplemental Information 1 Raw data All results of behavioral measures, EEG measures, and pot-dropping performance measures.

## Additional Information and Declarations

### Data Availability

The following information was supplied regarding data availability: The raw data is available in the Supplemental File.
